# Metastatic squamous cell carcinoma from hand skin causing small bowel obstruction: an unusual case presentation

**DOI:** 10.1186/1477-7819-12-166

**Published:** 2014-05-28

**Authors:** Ruixin Li, Zihua Chen, Qiaocheng Wen, Zhikang Chen

**Affiliations:** 1 Department of Gastrointestinal Surgery, Xiangya Hospital, Central South University, 87 Xiangya Road, Changsha, Hunan 410008, China

**Keywords:** Metastasis, Intestinal obstruction, Squamous cell carcinoma

## Abstract

The small bowel rarely suffers from metastatic tumors from outside the abdomen. Small bowel obstructions caused by the metastatic spread of squamous cell carcinoma (SCC) of the hand to the intestines are even rarer. A 71-year-old man with intermittent abdominal distension and pain for 4 months was diagnosed with partial bowel obstruction. The patient underwent a video capsule endoscopic examination; however, the patient was unable to pass the capsule, which worsened the abdominal distension. He was transferred to our department for acute intestinal obstruction, and an emergency exploratory laparotomy was performed. Intraoperatively, a tumoral stricture of the intestine at a distance of 150 cm from the ileo-cecum and dilation of the proximal bowel was found. The involved segment was resected, and ileo-ileal anastomosis was performed. The pathological sections confirmed the lesion to be a moderately differentiated SCC with whole bowel layer infiltration. Immunohistochemical staining showed positive expression of cytokeratin 5/6 and p63. The patient had an uneventful recovery. However, 6 months later, he was hospitalized again with intestinal obstruction. Reoperation was performed and revealed multiple metastases in the small bowel. He died 4 months later. In this unusual case, metastasizing SCC of the hand skin led to intestinal obstruction and poor prognosis. Therefore, follow-up procedures regarding intestinal spread should be performed in patients with SCC who present with abdominal symptoms.

## Background

Symptomatic intestinal or other intra-abdominal metastases from hand skin squamous cell carcinoma (SCC) are very rare. The majority of these metastatic lesions originate from the lung and esophagus [1-4]. Katz and colleagues reported a case of a renal transplant recipient who developed a small bowel obstruction caused by multiple organ metastases of skin SCC [5]. However, in our unusual and rare case, metastatic SCC of the hand skin metastasized to the small bowel and caused intestinal obstruction. Although the small bowel obstruction was successfully managed by laparotomy, the patient died 4 months later.

## Case presentation

**Figure 1:**
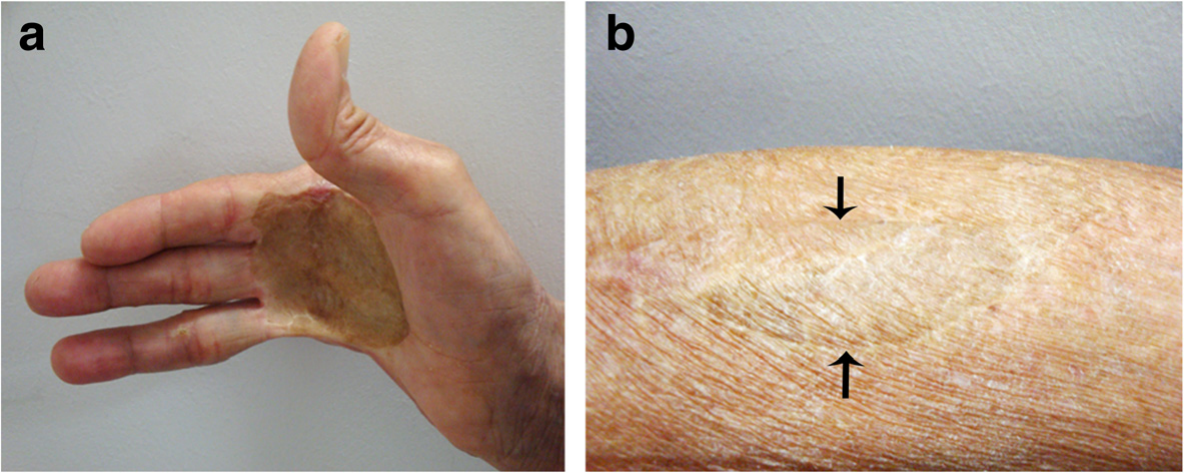
**Physical examination of the patient. (a)** Wide excision of the right little finger in the first operation and excision of the right palmar skin in the secondary operation. **(b)** Grafts using skin from the right forearm (arrowed) were performed in the secondary operation.

**Figure 2:**
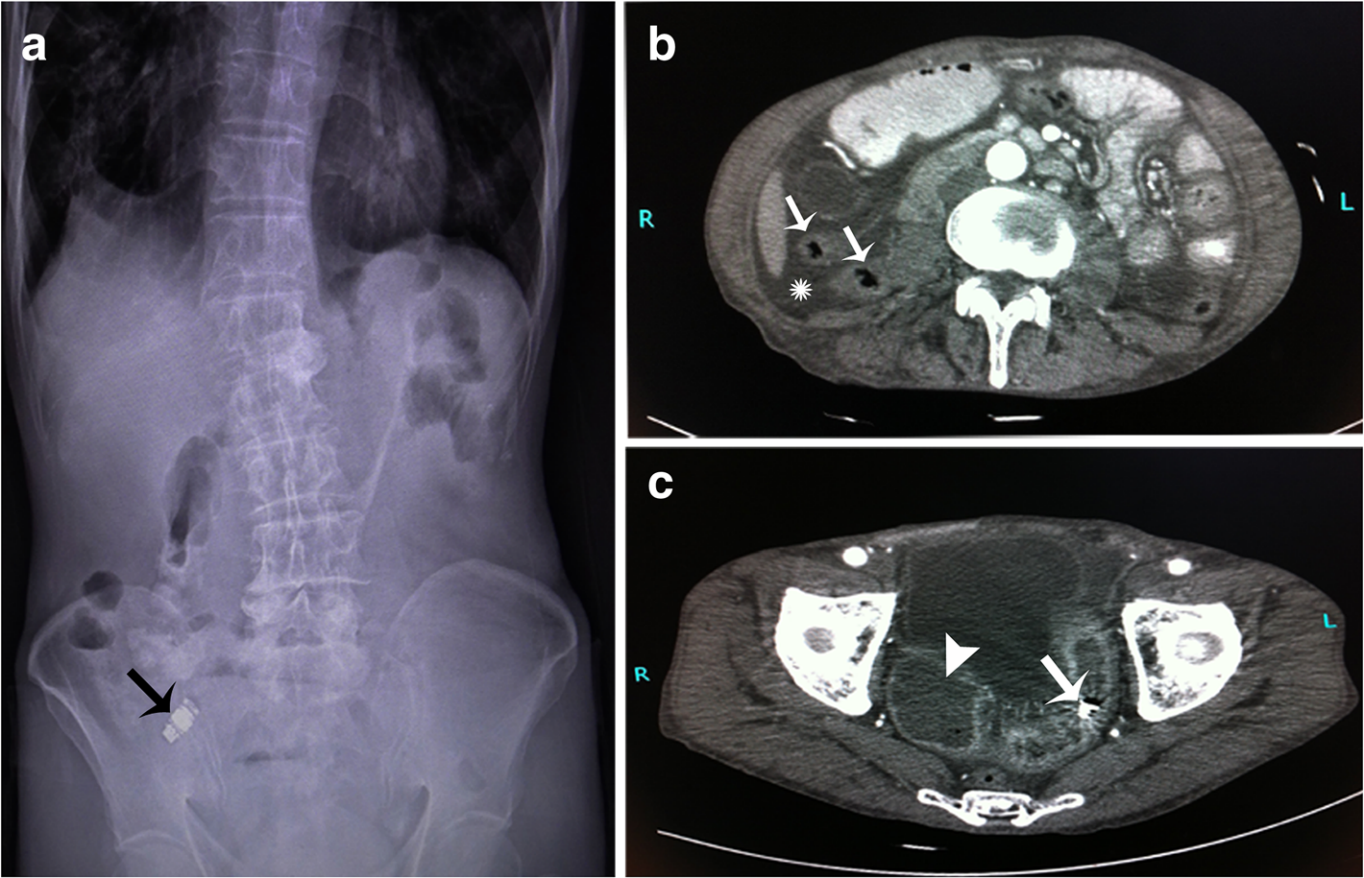
**Radiographic examination of the abdomen. (a)** Abdominal radiograph showed air-fluid levels and the endoscopy capsule (arrow) in the right lower quadrant. Oral contrast computed tomography showed **(b)** intestinal edema (arrows) and ascites (*) and **(c)** a high density object in the enteric cavity (arrow) and proximal intestinal dilation (arrowhead).

**Figure 3:**
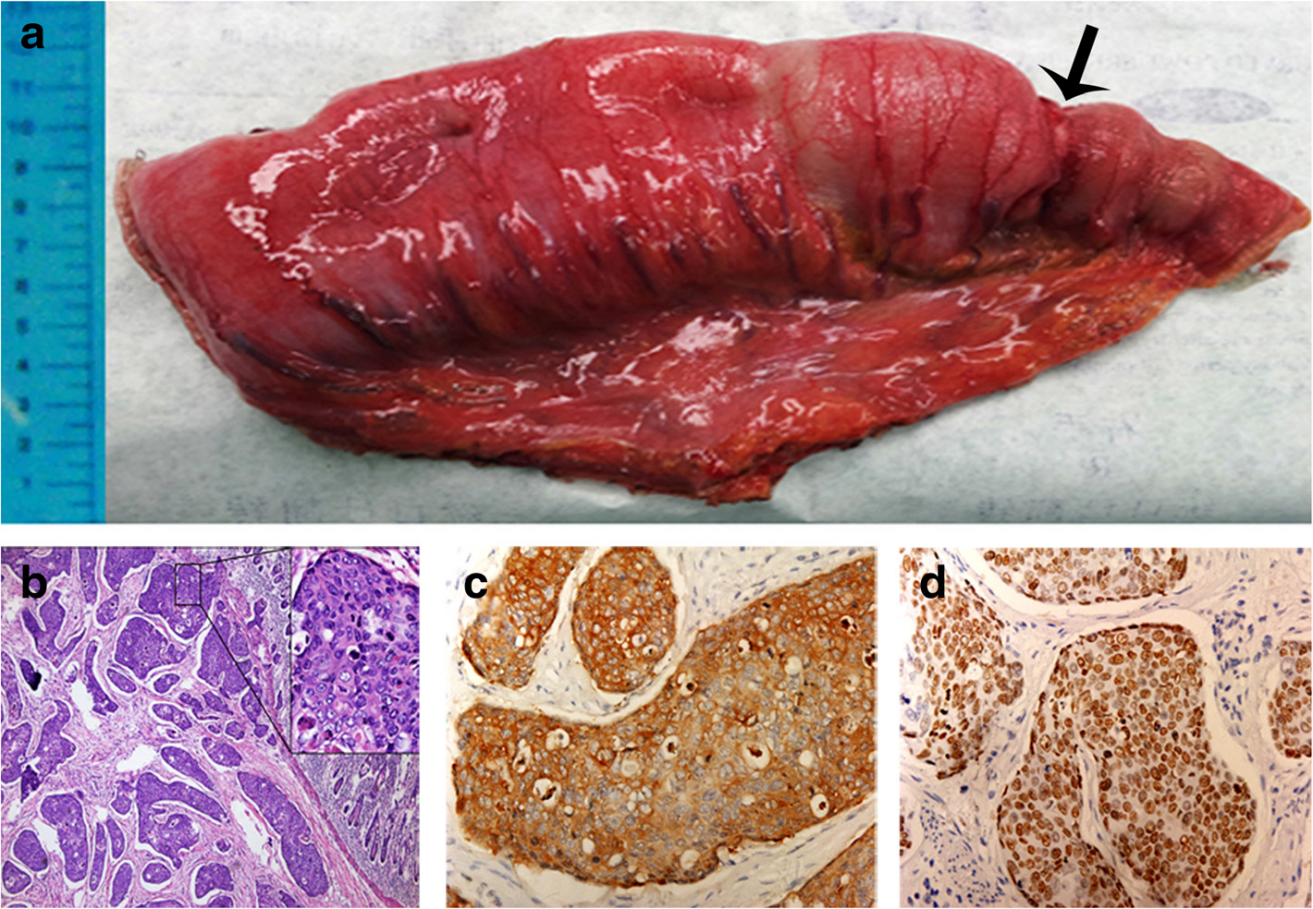
**Gross appearance and histological examination of the resected small intestine. (a)** The gross specimen showed a firm tumor stenosis (arrow) with an obvious dilation of the proximal bowel. **(b)** Hematoxylin and eosin stain, original magnification ×40, right upper ×200. **(c,d)** Streptavidin-peroxidase stain, original magnification ×200.

A 71-year-old man presented to a local hospital with a 4-month history of intermittent abdominal distension and periumbilical pain. The patient had bloating and loose stools but no nausea or vomiting. Eating aggravated the symptoms, whereas defecation relieved them. At the local hospital, capsule endoscopy was performed, and partial intestinal obstruction was diagnosed. The patient was transferred to our department because of capsule retention and worsening abdominal distension. He reported a 5-kg weight loss within the previous 4 months. His past medical history revealed that he had suffered from a 2 cm × 2 cm malignant skin ulcer on the right palm 10 years ago. At that time, he underwent surgery to remove the lesion with the aim of achieving a clear margin with a final range of 5 cm × 5 cm, which included resection of the little finger of his right hand (Figure 1a). The postoperative pathological analysis showed a T_2_N_0_M_0_ stage SCC with moderate differentiation. Unfortunately, he did not receive any other treatment except for one course of adjuvant chemotherapy with 5-fluorouracil and methotrexate. Three years ago, a wide surgical excision of recurrent skin lesions was performed (Figure 1a,b). The patient had no history of abdominal operation or nicotine addiction. There was no family history of cancer.Physical examination showed a well-developed man with malnutrition and anemic appearance. His vital signs were as follows: blood pressure, 96/64 mmHg; respiratory rate, 18/min; heart rate, 86/min; and body temperature, 37.2°C. Abdominal examination revealed moderate distention, high-pitched bowel sounds, and periumbilical and right lower quadrant tenderness associated with an impalpable mass. The rectal and genitourinary examinations were normal. Initial laboratory investigations revealed slight elevations in total bilirubin and direct bilirubin (0.15 mg/dl and 0.08 mg/dl, respectively). Other laboratory results, including the tumor markers carcinoembryonic antigen, carbohydrate antigen 199/125, and prostate specific antigen, were within their normal ranges. Plain abdominal radiographs found the video capsule and intestinal obstruction in the right lower quadrant (Figure 2a). The oral contrast computed tomography (CT) scan showed intestinal edema, ascites, and the video capsule in the enteric cavity (Figure 2b,c). Tumors in the esophagus and lung were not detected by gastroscopy or CT scan.Five days of conservative treatment alleviated the abdominal distension and pain. Unfortunately, capsule retention again aggravated the abdominal distension. In view of this circumstance, an emergency exploratory laparotomy was performed. Intraoperatively, a tumor circumferentially encroaching upon the ileum at a 150-cm distance from the ileo-cecal valve with dilation of the proximal bowel was present (Figure 3a). The endoscopic capsule could be palpated in the dilated lumen and was blocked by the stricture segment. There was no gross evidence of other tumors in the peritoneal cavity. A segmental bowel resection was carried out to remove the lesion. An end-to-end ileal anastomosis was adopted to re-establish the intestinal continuity. The pathological analysis revealed the tumor to be a moderately differentiated SCC (Figure 3b) with whole bowel layer infiltration and 1/10 lymph node metastasis. It was suggested that the obstruction of the ileum was caused by tumor invasion-associated stenosis. Immunohistochemical analysis showed positive reactions for cytokeratin (CK)-5/6 and p63 (Figure 3c,d), whereas caudal type homeobox 2, CK7, and villin proteins were not detected. These results were consistent with a SCC. The combined findings of the preoperative examinations and laparotomy, and the history of hand skin SCC led to a diagnosis of metastatic SCC of the intestine. Fourteen days later, the patient recovered and was discharged.

The patient refused to accept any other therapies after his discharge from the hospital. He developed a secondary small bowel obstruction and multiple SCC metastases in the jejunum and ileum 6 months after his first abdominal surgery. He underwent a second laparotomy, which revealed stenosis in multiple segments of the bowel caused by the invasion of metastases. Two severely separated segments were resected, and end-to-end anastomosis was performed. The patient was discharged on postoperative day 15 and died 4 months later at the age of 72 years.

## Discussion

Metastatic tumors of the small bowel account for 0.5% of all small intestinal malignancies. The majority of these metastases come from primary adenocarcinomas, whereas SCC is a very small proportion [6]. The diagnostic criteria for secondary small bowel SCC include primary SCC or history of excision of SCC from the lung or other organs. Histopathologically, secondary SCC generally presents as cancer invasion in the submucosal, muscular layer and serosa [1]. Secondary SCC does not usually involve the glandular epithelium [1].

Abdominal metastases from extra-abdominal cancer are not unusual, and tumor spread into the small bowel as a result of secondary SCC has been well reported [7-9]. Many extra-abdominal SCCs, such as those of the esophagus [1,2], lung [3,4], penis [10], ovary, pancreas and gallbladder [11], can metastasize to the small intestine. However, the lung is the most common extra-abdominal site for intestinal metastases [3,4,8,9]. Clinical manifestations of intestinal metastases include gastrointestinal obstruction, bleeding, perforation and intussusceptions. Small bowel obstructions caused by metastases are most unusual, and SCC arising from the palmar skin and metastasizing to the small intestine is extremely rare.

SCC of the skin commonly occurs in the elderly, especially those older than 70 years. The most common localizations are on photoexposed areas, such as the head, neck and backs of the hands. The ratio between photoexposed and non-photoexposed areas is 5:1 [12,13]. These localizations vary in men and women. SCC occurs 2.5 times more often on the nose area in women and six times more often on the ear in men. CK5/6 and p63 expression is a characteristic of SCC cells and is used to differentiate SCC from other types of neoplasms such as adenocarcinomas and stromal tumors [14].

Lymphatic spread is the common metastatic route of skin SCC [15]. Hand skin SCC metastasizes to the thoracic wall and lung through lymph nodes in the epitrochlear and medial axillary regions [16]. Esophageal and pulmonary SCC metastasizes to the small intestine via retrograde spread to the mesenteric lymph nodes or via the vertebral venous system [1-4,17]. This suggests a connection between the thoracic lymphatic or hematogenous system and the intra-abdominal region. Based on this potential connection, we infer that the palm SCC metastasized to the small intestine in our patient via the axillary and thoracic lymphatic or hematogenous system.

Tumor detection in the small bowel is difficult because of the liquiform nature of its contents. The liquiform contents of the small bowel can pass through even the tiniest opening, which allows a tumor to go undetected until it reaches an advanced stage or results in complications. The prognosis of patients with such tumors is very poor because of the few effective therapeutic approaches. In our unusual case, metastasizing SCC of the hand led to intestinal obstruction. The disease recurred and the patient died less than one year after the first laparotomy. Therefore, follow-up procedures regarding intestinal spread should be considered for patients with SCC who present with an acute abdomen or any other abdominal symptoms.

Current available approaches to detect lesions and metastases in the small bowel include endoscopy, contrast studies and CT scanning. Endoscopic procedures include small bowel endoscopy and video capsule endoscopy (VCE). Although VCE is relatively non-invasive, it is contraindicated in patients with swallowing disorders, implanted electromedical devices or known or suspected gastrointestinal obstruction, strictures or fistulas [18]. However, Mason and colleagues reported that VCE successfully detected subacute bowel obstructions for nine symptomatic patients with a prior negative diagnostic work-up, which led to surgical resection of the diseased bowel and cure of the patients’ signs and symptoms [19]. In our case, capsule endoscopy was performed by the local hospital despite the partial intestinal obstruction diagnosis. The patient was admitted in poor condition with recurring symptoms and had a lengthy stay in the local hospital; therefore, we did not use dual-balloon enteroscopy as a diagnostic tool because of the high possibility of intolerance and risk of perforation. As some authors consider gastrointestinal contrast with gastrografin to be the most useful test before operation [20], we selected oral contrast CT as an alternative. To our surprise, the CT scan showed that the video capsule was in the left pelvic cavity, which differed from the radiographic findings. This phenomenon may have been caused by intestinal movement.

Radiation and chemotherapy are generally used as adjuvant or palliative therapies in patients with hand skin SCC, especially for those with involved lymph nodes or distant metastases. In our case, the patient refused additional treatment after the first laparotomy and soon developed a recurrence.

Surgical resection should be considered for treating primary and metastatic SCC lesions and should include wide local excision of the tumor and lymph node-bearing mesentery. Some patients may require a second resection at a later date. The longest survival time without evidence of recurrent disease following resection of small bowel metastasis is 8 years [21]. In our case, the patient had a recurrence with the same symptoms 6 months after surgery. He underwent a second laparotomy, which temporarily alleviated the intestinal obstruction. Although patients with small intestinal metastatic disease have a dismal prognosis, surgery should be considered to relieve the intestinal obstruction. Patients would benefit from early diagnosis and quick and appropriate treatment.

## Conclusions

Hand skin SCC can metastasize to the thoracic wall and lung through lymph nodes in the epitrochlear and medial axillary regions. Skin SCC commonly metastasizes to the lung, liver, brain and bone, whereas intestinal obstruction caused by skin SCC metastases is an unusual presentation. SCC metastasizes to the small intestine through retrograde spread to the mesenteric lymph nodes or via the vertebral venous system. Although recurrences occur in most skin SCC cases, radical resection of the lesion is needed. Standardized chemotherapy after operation may improve the outcome for patients with distant metastasis of skin SCC.

## Consent

Written informed consent was obtained from the patient for publication of this case report and any accompanying images. A copy of the written consent is available for review by the Editor-in-Chief of this journal.

## Abbreviations

CK: cytokeratin; CT: computed tomography; SCC: squamous cell carcinoma; VCE: video capsule endoscopy.

## Competing interests

The authors declare that they have no competing interests.

## Authors’ contributions

RL participated in the operation, and drafted and completed the manuscript. ZC was involved in the operation and review of the literature. QW was involved in the acquisition of clinical pictures and pathological examinations. ZC conceived the study, participated in the operation, and supervised manuscript preparation. All authors read and approved the final manuscript.
